# Partnering to Improve Mentorship Capacity for Ugandan Reproductive Health Researchers: Program Description and Evaluation

**DOI:** 10.4269/ajtmh.23-0459

**Published:** 2023-11-20

**Authors:** Julie M. Buser, Ella August, Faelan E. Jacobson-Davies, Felix Bongomin, Edward Kumakech, Rachel Gray, Pebalo Francis Pebolo, Anna Grace Auma, Tamrat Endale, Yolanda R. Smith

**Affiliations:** ^1^ Center for International Reproductive Health Training, University of Michigan, Ann Arbor, Michigan;; ^2^ Department of Epidemiology, University of Michigan School of Public Health, PREPSS (Pre-Publication Support Service), Ann Arbor, Michigan;; ^3^ Department of Obstetrics and Gynecology, University of Michigan, Ann Arbor, Michigan;; ^4^ Department of Medical Microbiology and Immunology, Faculty of Medicine, Gulu University, Gulu, Uganda;; ^5^ Department of Nursing and Midwifery, Lira University, Lira, Uganda;; ^6^ Department of Reproductive Health, Faculty of Medicine, Gulu University, Gulu, Uganda

## Abstract

Mentorship is essential to health researchers in achieving their full potential and advancing public health. In most low-resource settings, there is a paucity of training on how to be a successful mentor. The Center for International Reproductive Health Training at the University of Michigan conducted and evaluated a workshop at two universities in Uganda for mentors of new reproductive health research grant awardees. The program aimed to strengthen mentors’ mentorship skills and to identify ways to foster institutional support for mentoring. Mentors rated their post-training skills using a 5-point Likert scale (not skilled to extremely skilled) immediately and 3 months after the training. Ten of 19 mentors who participated in the training completed the evaluation. The majority were 41 to 50 years old, male, midcareer faculty. Immediately after the training, mentors rated themselves (mean ± SD) highest in knowledge of research ethics (4.4 ± 0.5), fostering independence in mentees (4.3 ± 0.9), and understanding the benefits of mentoring (3.9 ± 1.1). Mentors felt least confident in fostering institutional change to support mentorship (3.3 ± 0.8), communication (3.5 ± 0.5), and overcoming adversity (3.5 ± 0.8). The two most important things the mentors learned were how to appreciate and manage diversity and how they can benefit from mentorship. Barriers to mentoring that persisted after the program ended included lack of time and institutional resources. Enhancing mentorship training opportunities will foster a generation of scientists who are more supported, skilled, and productive in research, leading to better reproductive and public health outcomes in their communities.

## INTRODUCTION

Mentorship is essential to health researchers in achieving their full potential and advancing public health, particularly for sexual and reproductive health and rights (SRHRs) research, where mentorship plays a big role in producing quality evidence to improve practice.^1^^,^^2^ In most low- and middle-income country (LMIC) settings, there is a paucity of training on how to be a successful mentor.^3^^,^^4^ Although many academic mentors in these settings have the necessary subject matter expertise, they may lack the knowledge, skills, and institutional resources needed to effectively mentor early-stage researchers.^5^ Strong mentorship training programs aimed at developing skills and practices that cultivate effective relationships could improve the depth and success of the LMIC global health academic workforce.^3^ Furthermore, when researchers grow professionally as a result of quality mentorship, their motivation to continue working with their institutions may be enhanced, reducing attrition and maintaining research efforts where they are most needed.^1^^,^^6^ In Uganda, as in many other low-income countries, academic mentorship has been identified as a key area for improvement to increase the quality and quantity of research produced and to support the development of the next generation of researchers.^7^ However, there are currently few formal training programs available to support academic mentors in this role.^7^^,^^8^

The Center for International Reproductive Health Training at the University of Michigan (CIRHT-UM) partners with medical, midwifery, and nursing faculty and students to strengthen research capacity and the provision of quality SRHR care by providing training, research opportunities, and mentorship.^9^ The CIRHT-UM’s faculty development program provides faculty in OB-GYN, midwifery, nursing, and collaborating departments with the skills, coaching, and professional networks to advance as SRHR educators, health providers, and researchers.^9^ Reproductive health research grants are awarded to faculty at partner institutions through a competitive application process to facilitate research experiences on SRHR topics of local relevance. Identifying and proposing mentors is part of the application process.

As part of the faculty development program, the CIRHT-UM conducted a training workshop in July 2022 designed specifically for the 31 mentors of the 48 new research grant awardees from Gulu and Lira Universities and their affiliated hospitals in northern Uganda to strengthen their mentorship skills and to identify ways to foster institutional support for mentoring. To address the gap in formal mentor support, a training program was developed and implemented by the CIRHT-UM to strengthen the capacity of research mentors in Uganda. The overarching learning objectives were to 1) demonstrate understanding of key SRHR research mentorship competencies, 2) define the skill set required for effective mentoring, and 3) identify opportunities for strengthening institutional capacity to support mentors. The purpose of this paper is to describe the CIRHT-UM mentoring training and evaluate the response to a full-day in-person training in Uganda for mentors of new research grant awardees.

## MATERIALS AND METHODS

### Participants.

Mentors in this in-person mentoring training program were healthcare research mentors from Gulu and Lira Universities and their affiliated hospitals in northern Uganda. An informal verbal needs assessment was conducted among potential mentors prior to developing the training materials to identify their mentoring needs and gaps. Members of the authorship team had individual discussions with several mentors about confidence in their mentorship skills, challenges with research mentorship, and what they viewed as areas for improvement.

Local mentors were identified by Ugandan CIRHT-UM research grant recipients and described in their proposals. Mentors were chosen because they were senior faculty members or clinicians and experienced researchers who agreed to guide and support grant awardees throughout the research process. Research grant recipients were encouraged to identify mentors who would help them define research goals, share knowledge, provide encouragement, and give feedback on scientific work in a constructive and timely manner.^10^ Of the 30 local mentors invited, 19 attended the training.

### Overview of training.

**Figure 1.**
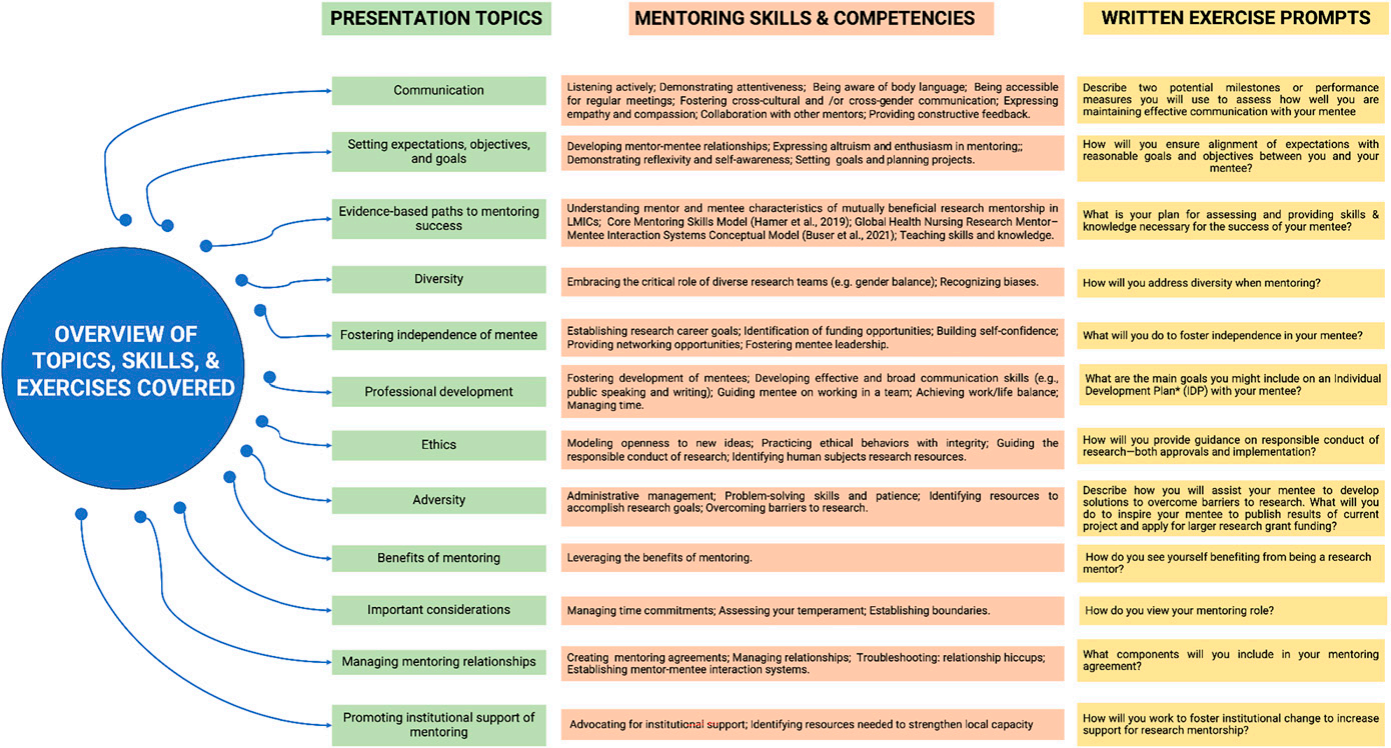
Overview of topics, skills, and exercises covered in the CIRHT-UM training for mentors of new sexual and reproductive health grant awardees in Uganda in July of 2022 (adapted from Hamer et al.^5^ and the NIH^11^). *An individual development plan (IDP) outlines steps to improve performance and meet career goals.^12^ CIRHT-UM = The Center for International Reproductive Health Training at the University of Michigan; LMIC = low- and middle-income country.

The full-day, on-site, face-to-face training consisted of 13 modules including assessing mentoring skills, independence, diversity, communication, professional development, mentoring relationships, ethics, fostering institutional change, and benefits of mentoring (Figure 1). The agenda with presentation topics and proposed format was shared with Ugandan primary investigators and research team members for their evaluation and feedback about 1 month before the training. The training was developed and led by J. M. B., a staff member from the CIRHT-UM with expertise in scientific research and mentoring. The bulk of the training was modeled on the adapted framework for global health research mentoring competencies and skills outlined by Hamer and others^5^ in a supplement on mentoring in LMICs to advance global health published by the *American Journal of Tropical Medicine and Hygiene*.^13^ In addition, several NIH mentoring program materials,^11^ the *Global Health Nursing Research Mentor–Mentee Interaction Systems Conceptual *Model,^14^ and the American Speech-Language-Hearing Association^15^ provided content for topics covered in the training. PowerPoint presentations, lasting between 30 and 45 minutes each, either included topics for the mentors to learn, such as advocating for institutional support, or were topics that mentors need to model or teach for mentees. Two local Ugandan research coordinators from the CIRHT-UM’s partner institutions served as facilitators for the training and assisted with its organization. The training program was designed to be interactive and participatory through discussions and experience training, with a set of newly created written workbook exercises covering the fundamentals of effective mentoring (Figure 1). After each mentoring topic was presented, mentors answered the workbook exercises individually, then discussed responses as a group for between 15 and 30 minutes. Mentors had the opportunity to pose questions for discussion to each other and the trainer. Training was evaluated using two time-staggered post-test surveys.

### Data collection and analysis.

Data were collected anonymously using Google forms distributed via E-mail through post-training surveys. The first post-training evaluation was sent to all mentors 1 week in advance of the training and was completed immediately after training. Additional post-training evaluations were sent 3 months after the training by both E-mail and a WhatsApp group by local coordinators; both surveys had the same questions to be completed within 2 weeks with reminders sent every other day. WhatsApp was used because it was preferred by the majority of local mentors because of internet connectivity issues accessing E-mail. Consistent with other mentoring training studies, we selected the 3-month follow-up time frame to give participants time to reflect on and apply their training with current mentees.^16^^–^^18^ The survey collected demographic information (including age, sex, title), familiarity with training topics (e.g., effective communication, independence), and self-assessment of mentoring competencies (e.g., setting expectations, objectives, and goals) (Supplemental Material). Mentors rated their mentoring skills using a 5-point Likert scale (not at all skilled to extremely skilled). Finally, the mentors were asked to provide general comments about the training using open-ended feedback. Mentors’ responses were analyzed using descriptive statistics, the Wilcoxon rank sum (Mann-Whitney) test, and two-sample *t* tests via STATA V 17.0.

### Ethical considerations.

This study received exemption from the relevant institutional review boards at the University of Michigan (HUM00203642) and universities in Uganda (GUREC-2022-252). Informed consent was obtained from all mentors before having them complete training evaluations. We ensured mentor confidentiality by administering anonymous surveys. Responses were nonidentifiable and stored in a secure drive to protect mentors’ confidentiality.

## RESULTS

### Sociodemographic characteristics.

Of the 30 people who were invited to the academic mentoring training, 19 participated in the program. Ten of the 19 mentors who attended the training workshop completed the evaluation form in the immediate post-period, and 11 mentors responded in the 3-month post-training period. Of the survey respondents, the majority were 41 to 50 years old, male, mid-career lecturers with advanced degrees (PhD, MMed). The survey respondents reflect the population of specialist physicians, nurses/midwives, and faculty members who constitute the workforces in the two universities and their affiliated teaching hospitals.

### Self-rated skills in mentoring.

Immediately after the training, mentors rated themselves (mean ± SD) most skillful in ethics (4.4 ± 0.5), independence (4.3 ± 0.9), and understanding the benefits of mentoring (3.9 ± 1.1) (Table 1). Conversely, mentors felt least skillful in fostering institutional change to support mentorship (3.3 ± 0.8), communication (3.5 ± 0.5), and adversity (3.5 ± 0.8) (Table 1).

**Table 1:** Self-rated mentoring skills reported by mentors immediately after the training workshop and 3 months after the workshop using a Likert scale

Self-rated skills	Immediately after intervention		3 Months after intervention		
	Mean	SD	Mean	SD	*P* value
Maintaining effective communication	3.5	0.5	3.5	0.7	0.9
Aligning expectations	3.8	0.6	3.5	0.7	0.4
Assessing and providing skills	3.7	0.7	3.9	0.7	0.5
Addressing diversity	3.6	1.1	3.5	0.8	0.9
Fostering independence	4.3	0.9	3.7	0.8	0.2
Promoting professional development	3.8	0.8	4.4	0.8	0.1
Promoting integrity and ethical conduct	4.4	0.5	4.2	0.6	0.4
Overcoming adversity	3.5	0.8	3.4	0.7	0.7
Understanding the benefits of mentoring	3.9	1.1	4.1	0.8	0.7
Maintaining successful mentoring relationships	3.6	0.5	3.9	0.5	0.2
Fostering institutional change to promote mentoring	3.3	0.8	3.5	0.5	0.6

Likert scale of skill level (1 = lowest and 5 = highest). *P* values were calculated using a two-sample *t* test.

In the 3-month postsurvey, the highest self-rating averages (mean ± SD) were in promoting professional development (4.4 ± 0.8), integrity and ethical conduct (4.2 ± 0.6), and understanding the benefits of mentoring (4.1 ± 0.8) (Table 1). Mentors felt least skillful in overcoming resource limitations and other sources of adversity (3.4 ± 0.7) and fostering institutional change (3.5 ± 0.5) (Table 1). We compared the self-assessments using two-sample *t* tests, and the difference in the mean responses for each self-reported skill were nonsignificant. We also compared the difference between immediate post responses and 3-month post-training responses, but no significant differences were found and the study did not include the rigor necessary to statistically test whether there was no change between time periods.

### Individual strengths and areas for improvement as a mentor after the training.

The mentors responded to questions about their strengths and areas for improvement as a mentor after the training. The two highest-scoring strengths among the mentors were being flexible (*n* = 2) and knowledgeable (*n* = 2). Meanwhile, the three most common areas for improvement among the mentors were being more flexible (*n* = 3), becoming more knowledgeable (*n* = 2), and becoming willing or able to devote time to developing others (*n* = 2).

### Barriers to mentoring.

Ten mentors in the training program responded to questions on their perceptions of the institutional and individual barriers to mentoring after the training ended. Table 2 shows that the barriers perceived by most of the mentors were individual barriers and they lacked time and formal training in mentoring.

**Table 2:** Perceived institutional level and individual barriers to mentoring

Barriers	Proportion of mentors who responded to the question (*n* = 10)
Institutional level barriers	
Lack of acknowledgment from institution for being a mentor	0.2
Lack of resources	0.2
Lack of time	0.2
No formal training programs	0.2
No funding	0.2
Individual barriers	
Lack of time	0.5
No formal training	0.4
Power differential between mentor and mentee	0.1

### Mentor knowledge gained and general comments.

Mentors were asked about the most important thing they learned from the mentoring workshop. The most common responses were how to appreciate and manage diversity and how they could benefit from mentoring.

The mentors in the training workshop responded to an open-ended question on their plan for one thing they intend to incorporate into their mentoring activity going forward. The findings presented in Table 3 show that three of the nine mentors who responded to the question planned to create a formal mentoring plan or agreement. Finally, the mentors were asked to provide general comments about the mentor training. They were asked “What is the most important thing you learned from the mentoring workshop?” From the responses, we identified six common themes: appreciate and manage diversity (40%), how to benefit from mentorship (20%), professionalism in mentorship (10%), building relationships and communication (10%), focused objective engagement (10%), and learning to be available (10%).

**Table 3:** Additional mentoring activities that mentors plan to incorporate into practice

Activities mentors plan to incorporate moving forward	Proportion (*n* = 9)
Have a formal mentoring plan or agreement	0.30
Acquire additional mentorship skills	0.10
Focus on career development	0.10
Continuous research for publication	0.10
Intentionality	0.10
Give full support to mentee in reaching their goals	0.10
Mobilize existing resources for research	0.10

## DISCUSSION

The purpose of this paper is to describe and evaluate the in-person CIRHT-UM mentoring training in Uganda for mentors of new research grant awardees. Mentorship training significantly improved familiarity with mentoring strategies for mentors who responded to our evaluation, and mentorship self-rated skills stayed relatively consistent immediately after and 3 months after training. The results of this study suggest that the training program was effective in achieving its learning objectives to 1) demonstrate understanding of key SRHR research mentorship competencies, 2) define the skill set required for effective mentoring, and 3) assess opportunities for strengthening institutional capacity to support mentors. The mentors reported that the workshop exercises were helpful and provided an opportunity for peer learning, support, and feedback from the trainer.

Furthermore, this study indicated mentors were also able to identify their own mentoring strengths immediately and several months after the training. Mentors were able to identify where they excelled as a mentor and where they needed to make adjustments. In addition, consistency across self-rated skills immediately after and 3 months after the training indicates some knowledge retention. Although we did not specifically test for change in self-skills assessments, we did not detect a change across a 3-month period, which may indicate consistent knowledge across time. After 3 months of putting into practice what was learned in the training program, mentors rated themselves better at promoting professional development and maintaining successful mentoring relationships but lower in fostering independence. Although significant findings were not detected, the small sample size limited the power of the statistical test; therefore, further testing, more rigorous survey methodology, and larger sample size are needed to determine if mentors retained knowledge for a period after training.

In Uganda, most lecturers have adequate training and experience in basic sciences, clinical medicine, and epidemiology and are able to mentor junior healthcare providers.^7^ The main challenges are related to limited mentoring skills, lack of time to meet, and lack of logistical support.^7^ In Uganda, local mentors are also burdened with clinical care, teaching, institutional development, advocacy, and research demands.^19^ A collaborative approach by institutional leadership and academic mentors is needed to design innovative ways of strengthening mentorship capacity in Uganda.

Our findings are in line with those of other mentoring training programs conducted in LMICs. In an evaluation of intensive workshops to train investigators conducting scientific research in several LMICs on mentoring, Gandhi and others^3^ found that common barriers included a lack of a culture of mentoring, time constraints, a lack of formal training, and a lack of recognition for mentoring. Access to mentorship training at the institutions remains low; however, short 1- or 2-day workshops, as this study demonstrates, are effective in training several mentors at a time. As Bennett et al.^8^ discussed, time is a larger barrier to mentoring in LMICs, such as Uganda, because there are fewer faculty trained as mentors and available to take on such roles. As more researchers are trained to mentor, the time burden will lessen. Institutions, and those who fund them, will need to continue to support mentorships and foster relationships with younger researchers. Furthermore, institutional support for mentoring training could be a way to mitigate the emigration of highly trained professionals from LMICs. This program differed in its context in Uganda and in that each mentor was specifically supporting a seed grant–funded PI with a multidisciplinary team (proposal requirement) with a strict time line. Therefore, these mentors had active, ongoing mentoring activities with opportunities to reflect on areas they wished to further develop and to practice new skills.

### Strengths and limitations.

The strengths of this training program include the focus on practical skills that can be immediately applied in mentors’ mentoring and research activities. The training program was tailored to the specific needs of the mentors, which ensured its relevance and effectiveness. Because the sample size was relatively small, generalizability of the findings is limited. In addition, the evaluation lacked a pretraining assessment for comparison with the post-training survey. Furthermore, the post-training survey had a poor proportional response rate. A potential reason for this could be limited access to reliable internet service. Perhaps distributing a hard copy version immediately after the training would have been more effective in gathering responses from a larger sample of training attendees. Most of the mentors were more experienced researchers, and this could have affected their preexisting skills and resources and, ultimately, the study results. Furthermore, the use of anonymous data may have limited our ability to perform more in-depth statistical analysis, including paired comparisons. Performing a formal needs assessment prior to developing the training would allow for an even more targeted approach to the training. A long-term follow-up to assess the sustainability of the self-assessed improvements observed in the mentors’ knowledge and skills is planned for the end of the collaborative CIRHT-UM project in November 2023.

### Lessons learned.

Including a balance of males and females has been shown to offer numerous benefits for this type of training^20^; however, we had only one female mentor in our program. Women in academia and reproductive healthcare need more mentorship and leadership training to advance their careers because of systemic barriers, such as organizational and structural gender inequalities.^1^^,^^21^ Mentors noted addressing diversity was one of the lower-rated skills, indicating mentors are likely aware of these discrepancies but do not have the tools and resources to overcome them. Having more female mentors in academics in LMICs is essential for promoting gender equity and more diversity in the academic sphere. Female mentors can also provide a valuable perspective on issues affecting women in academia, such as work-life and caregiving balance, sexual harassment, and discrimination.^22^^,^^23^ Ultimately, increasing the number of female mentors in LMICs can contribute to a more diverse and inclusive academic community and promote gender equality in education and research. In future projects, we will take steps to actively recruit females by encouraging group mentorship opportunities and holding discussions about navigating work-life balance.

On another note, we noticed that the majority of the mentors were either senior faculty or supervisors of the researchers. This implies that mentors must be a senior person or a supervisor, neglecting the role of peers who have been shown to be better mentors.^24^ People tend to learn easily and freely from peers.^24^ Even though there are challenges of bilateral learning and the risk of one peer taking over the mentoring relationship, peer mentorship has shown benefits such as facilitating open communication and coordination, fostering learning, encouraging self-learning, and reducing the risk of role conflict in the mentoring relationship.^25^ Including peer mentorship in research mentoring programs may foster a positive culture of research, improve research output, and promote evidence-based reproductive health practice.

## CONCLUSION

The findings of this study can inform the development of similar training programs in other LMICs, as well as provide insights into the challenges and opportunities of mentoring academic mentors in these contexts. Enhancing mentorship training opportunities will foster a generation of scientists who are more supported, skilled, and productive in research, leading to better reproductive and public health outcomes in their communities. To advance reproductive health, we encourage healthcare and academic institutions to dedicate time and resources for formal research mentorship training while addressing identified barriers.

## Supplemental files

10.4269/ajtmh.23-0459 Supplemental Materials
