# Supplementary material for: Partnering to Improve Mentorship Capacity for Ugandan Reproductive Health Researchers: Program Description and Evaluation
Source: Am J Trop Med Hyg. 2023 Nov 20;110(1):188–93. doi: 10.4269/ajtmh.23-0459 (PMC10793019; doi:10.4269/ajtmh.23-0459)
Supplement: Supplemental Materials [file tpmd230459.SD1.pdf]

Questions posed during the mentoring training in Uganda. Distributed via email and WhatsApp immediately and three months after the training.

Do you have any formal experience in mentoring? Yes/no

If yes, for how many years? <1, 1-5, 6-10, >10

If yes, what is your current number of mentees? List options or fill in number

Do you have formal training on mentoring? Yes/no

If yes, for how many hours did you participate in formal training on mentoring? List options or fill in number

Have you been named a mentor on an externally funded grant in the past? Yes/no

Please rate how skilled you feel you are in each of the following areas: Likert scale response 1-5 not all skilled to extremely skilled

- Maintaining effective communication
- Aligning expectations with reasonable goals and objectives
- Assessing and providing skills and knowledge necessary for success
- Addressing diversity
- Fostering independence
- Promoting professional development
- Promoting professional integrity and ethical conduct
- Overcoming resource limitations and other sources of adversity
- Understanding the benefits of mentoring
- Mentoring relationships
- Fostering institutional change

What are your individual strengths as a mentor? Choose all that apply (from https://www.asha.org/students/mentoring/excmentor/)

- Good listener
- Flexible
- Value diversity of perspectives
- Knowledgeable
- Nonjudgmental
- Able to give constructive feedback

- Honest and candid
- Able to network and find resources
- Successful in career
- Willing/able to devote time to developing others
- Eager to learn
- Other (fill in the blank)

What areas of improvement do you have as a mentor? Choose all that apply

- Become a good listener
- Be more flexible
- Value diversity of perspectives
- Become more knowledgeable
- Be less judgmental
- Be able to give constructive feedback
- Be more honest and candid
- Become able to network and find resources
- Be more successful in career
- Become willing/able to devote time to developing others
- Be eager to learn
- Other (fill in the blank)

What institutional level barriers to mentoring do you face? Choose all that apply

- Lack of resources
- No formal training program
- Lack of time
- No funding
- Lack of acknowledgement or recognition
- Poor planning
- Lack of incentive
- Other (fill in the blank)

What individual barriers to mentoring do you face? Choose all that apply

- Lack of time
- Lack of knowledge
- No formal training

- Gender
- Mismatched expectorations between mentor and mentee
- Power differential
- Personality
- Other (fill in the blank)

What is one thing you plan to add to your mentoring activities going forward? Open ended response

What is the most important thing you learned from the mentoring workshop? Open ended

Please list any additional mentoring training that would be useful to you. Open ended

What is your age?

What is your gender? Male, female

What is your title? Choose all that apply

- Assistant Lecturer
- Lecturer
- Assistant Professor
- Associate Professor
- Professor
- Nurse
- Midwife
- Physician
- Other (fill in the blank)

What type of advanced degrees do you hold? Choose all that apply

- Nursing Diploma
- Bachelor of Nursing
- Bachelor of Midwifery
- Master of Nursing
- Master of Midwifery
- MBChB
- MSc
- MMed
- PhD

- Other (fill in the blank)
